# Is the head-mounted laser an appropriate tool to measure cervical movement across three planes?

**DOI:** 10.1080/23335432.2026.2624302

**Published:** 2026-01-29

**Authors:** D. J. English, N. Weerakkody, A. Zacharias, R. A. Green, M. de Noronha, C. Hocking, A. Kumar, X. Li, R. Rico Bini

**Affiliations:** a Department of Rural Allied Health, La Trobe Rural Health School, La Trobe University, Bendigo, Victoria, Australia; b Department of Rural Clinical Sciences, La Trobe Rural Health School, La Trobe University, Bendigo, Victoria, Australia; c Department of Rural Health, The University of Melbourne, Bendigo, Victoria, Australia; d Department of Mathematical and Physical Sciences, La Trobe University, Bendigo, Victoria, Australia

**Keywords:** Neck, assessment, clinical, proprioception

## Abstract

The head-mounted laser is commonly used in clinical proprioceptive tests, but its ability to measure movement across three planes of motion has not been investigated. Therefore, this study evaluated the head-mounted laser against a validated gold standard multi-sensor inertial measurement unit (IMU) (Xsens^TM^) in measuring cervicocephalic movement across three planes. Fourteen healthy adults (seven males and seven females) performed six repetitions each of active cervical flexion, extension, lateral flexion and rotation, whilst instructed to maintain the laser beam within a bullseye corresponding to 4.5° of movement. Primary plane means Xsens^TM^ range of motion (ROM) was used to evaluate if movement remained less than 4.5°. During flexion, extension and rotation movements, mean ROM in the primary plane using Xsens^TM^ remained within the 4.5° threshold (2.97°−3.57°), indicating that the head-mounted laser corresponded with the IMU (*p* ≤ 0.01). However, Xsens^TM^ mean lateral flexion movement reached 11.34° and 12.10° for left and right lateral flexion, indicating poor correspondence (*p* = 0.95 and 0.97). The head-mounted laser is appropriate for clinical movement assessment in the sagittal and transverse planes but not recommended for the coronal plane. Other devices should be considered for complete clinical assessment of cervical position sense.

## Introduction

Proprioception is our sense of body position, movement and force (Riemann and Lephart 2002). Evaluation of cervicocephalic proprioception is important because it is often impaired in people with neck pain (de Vries et al. 2015; Stanton et al. 2016). A range of tests have been designed to measure proprioception, utilising varying equipment and methodology (Hillier et al. 2015).

The head-to-neutral joint position error test (Revel et al. 1991) is commonly used to measure sense of position (Peng et al. 2021). The blindfolded participant is required to relocate their head to their neutral position following an active cervical movement, and the position error is the difference between the initial and final positions (Revel et al. 1991). Reviews on the validity of the head-to-neutral test have found generally higher position error in people with neck pain versus controls (de Vries et al. 2015; Stanton et al. 2016). Cervical position errors greater than 4.5° are commonly applied as the threshold of proprioceptive impairment (Revel et al. 1991), but the differences in cervical position error between controls and people with neck pain are small (Roren et al. 2009; Dugailly et al. 2015). In practical terms, one study found an 80% chance that neck pain participants reposition the head outside the 4.5° limit and healthy participants reposition within it, indicating that there is some overlap between groups (Roren et al. 2009). Additionally, there is low correlation in cervical position error between repositioning movements (e.g. flexion-extension versus lateral flexion versus rotation) (Swait et al. 2007) and people with whiplash may show proprioceptive impairments in one plane but not another (i.e. transverse versus sagittal) (Treleaven et al. 2003). Given that rehabilitation requires the identification of specific impairments, it is essential that all three planes of motion be evaluated (Röijezon et al. 2015). The small differences between controls and clinical populations combined with the need to evaluate tri-planar movement necessitates the use of highly accurate equipment.

**Figure 1.**
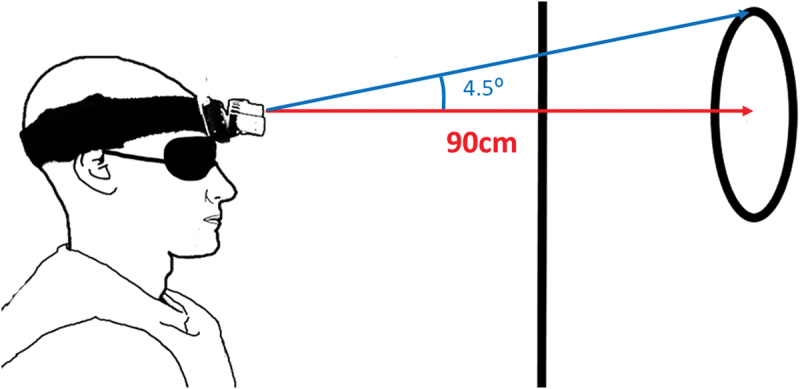
The head-mounted laser method for the head-to-neutral position error test. The seated and blindfolded participant is asked to perform an active cervical movement and then reposition the head to their initial ‘neutral’ position. Error is measured in centimetres then converted to degrees, whereby 4.5° is considered the threshold for healthy individuals.

Conclusions drawn from systematic reviews showing position error differences between people with neck pain and controls are based on many studies utilising laboratory-based equipment such as ultrasound-based motion analysis and electromagnetic devices (de Vries et al. 2015; Stanton et al. 2016). However, there are practical restrictions on the type of equipment used to measure cervical movement in the clinical setting. Traditionally, clinicians measure cervical active range of motion (AROM) using alternative tools such as the Cervical Range of Motion (CROM) device, goniometers and inclinometers (Lemeunier et al. 2020). For proprioceptive tests, a head-mounted laser and bullseye is commonly employed to assess position error, where the distance of the laser beam from the bullseye reflects magnitude of error (Revel et al. 1991) (Figure 1).

Compared to laboratory equipment, the laser method has practical advantages regarding affordability and transportability, but assumptions based on highly accurate tools should not be extrapolated to the clinical setting without further considering the accuracy of the method involved (de Koning Ch et al. 2008). Studies investigating the reliability and validity of the head-mounted laser have only focussed on sagittal and transverse plane movements (English et al. 2022). Using an intraclass correlation coefficient (ICC) grading system of <0.5 = poor, 0.5–0.75 = moderate and >0.75 = good (Portney and Watkins 2009), reliability for the head-to-neutral test using the laser method is moderate for head rotation (Burke et al. 2016) and moderate-good for flexion-extension motions (Zito et al. 2006; Pinsault et al. 2008; Roren et al. 2009; Dugailly et al. 2015; Amiri Arimi et al. 2018; Gonçalves and Silva 2019). Regarding validity, one study found high correlation of the head-mounted laser with a three-dimensional ultrasound-based technique, but only cervical rotation was evaluated (Roren et al. 2009). No studies have investigated lateral flexion movements across the coronal plane (English et al. 2022).

Theoretically, the head-mounted laser has potential limitations in measuring movement across the coronal plane (i.e. cervical lateral flexion) because movements of the head and neck occur in both primary and associated planes (Ferrario et al. 2002; Sforza et al. 2002). Associated planar movement is negligible for cervical flexion-extension (less than 5° through full range of motion), but during lateral flexion, there is significant associated rotation occurring in the opposite direction (i.e. lateral flexion to the left involves rotation to the right) (Ferrario et al. 2002). This is normal and expected given the oblique orientation of the cervical facets necessitating head movement across more than one plane of motion (Cook et al. 2006). The amount of contralateral rotation is variable (Ferrario et al. 2002). However, it is unclear whether this variability is between or within individuals.

Given the theoretical limitations of the head-mounted laser in measuring coronal plane movement and absence of previous reports, the primary aim of this study was to evaluate the efficacy of the head-mounted laser in measuring cervical movements against a validated gold standard, Xsens^TM^. We hypothesise that the laser will be unable to detect all coronal plane head movement (cervical lateral flexion) that is measured by Xsens^TM^. Variability of movement between participants and within participants will also be evaluated to investigate within-session movement variability.

## Methods

### Participants

Fourteen healthy volunteers aged 18–50 years were recruited from the local community (seven males, seven females; mean age 30.6 years (SD 10.4)). As the study aimed to evaluate the efficacy of a measurement tool (the head-mounted laser), and not identify impairments in a clinical population, healthy participants were considered ideal for the study. There are limited data to calculate sample size for this type of study, so a convenient sample was obtained through a concurrent study (English et al. 2023). Participants were recruited through advertisements at the University and screening was completed by the primary researcher, a physiotherapist (DE) using subjective assessment. People were excluded if they had neck pain in the previous 3 months, injury to the head or cervical spine, neurological disorder, impairment of the vestibular system (including vertigo), systemic disease or pregnancy. Human ethics was approved for this study (HEC20024), which conformed with the Declaration of Helsinki. Informed consent (written and verbal) was obtained for each participant, and each was designated a participant number to ensure anonymity during data analysis.

### Equipment

Xsens^TM^ is a wireless IMU motion capture system that tracks movement of the body in three dimensions. A systematic review found that IMUs demonstrate good joint measurement validity when applied to the neck region (Poitras et al. 2019). Xsens^TM^ was selected as the gold standard for this study because it demonstrates high coefficient of correlation (0.84–0.98) against optoelectronic systems, with accuracy within 0.5°–1° when measuring simple movements of the neck (Robert-Lachaine et al. 2017). Nine wireless motion tracking sensors, sampling at 100 Hz, were attached to the body (left and right forearm, left and right upper arm, left and right scapula, sternum, sacrum and head) using elastic straps, according to manufacturer guidelines (MVN Biomech Awinda, Xsens Technologies BV, Enschede, the Netherlands). Xsens^TM^ software calculates localised joint movement by using sensor reference points to separate the body into segments. Movement of the head and neck is calculated as upper and lower cervical movement, and for the purpose of this study, they were combined to create a sum of total cervical movement.

**Figure 2.**
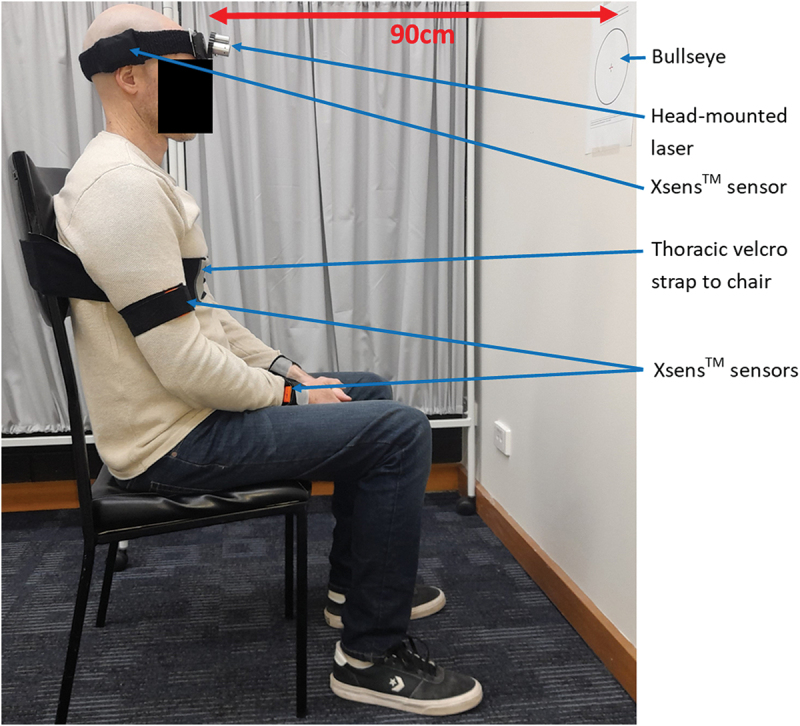
Experimental set up with participant positioned 90 cm from the bullseye, secured to a chair and fitted with Xsens^TM^ and the head-mounted laser.

A standard head-mounted laser (Senhang SH-6652) was attached to the participants head and directed towards a bullseye target, placed 90 cm anteriorly (Figure 2). The bullseye had a radius of 7.08 cm, corresponding to a 4.5° range of head motion, chosen to reflect the clinical threshold for the head-to-neutral position error test (Revel et al. 1991). An iPad camera (iPad OS 16.7) was used to record the procedure for visual inspection.

### Experimental procedures

Each participant completed one testing session in a university laboratory. The same operator conducted all trials to control for inter-rater reliability. Once fitted with the laser and Xsens^TM^ as per software system protocols, the participant was seated in a high-back chair (level with T4 vertebra at minimum) in which their head was positioned 90 cm from a forward-facing wall. To eliminate trunk movement, the participant’s trunk was secured using a Velcro strap placed under the axilla (English et al. 2023) (Figure 2). The person was asked to close their eyes, assume their natural posture and the bullseye was positioned to centre over the projected laser beam.

The participant was advised to open their eyes and perform a familiarisation of six repetitions of full active cervical flexion, extension, rotation (left and right) and lateral flexion (left and right). No additional warm-up movements were performed. For the actual testing, the participant was instructed to perform the same movements but deliberately move the laser beam from the centre (neutral position) to the edge of the bullseye (final position) and then return to the initial position, with their eyes open. Movements were standardised using the following instruction provided to each participant: ‘*we’re going to measure the amount to which you can move the head whilst maintaining the laser within the bullseye, moving in the same way as before (i.e. familiarisation). You may move slowly if required’*. The final position in movement execution was determined when the participant reversed direction in returning their head to the initial position.

The order of movement was randomised (random.org) and a total of six consecutive repetitions were performed for each direction. Using Xsens^TM^, cervical ROM (expressed in degrees) was recorded across the three cardinal planes (sagittal, transverse and coronal) for every trial (one primary plane and two associated planes).

Trials were only included if the laser beam remained within the bullseye and were discarded (not analysed) if it exceeded the radius, as evaluated retrospectively by a single researcher using video analysis captured by the iPad camera. Therefore, the maximum amount of motion detected by the laser beam was 4.5°.

### Data analysis

**Figure 3.**
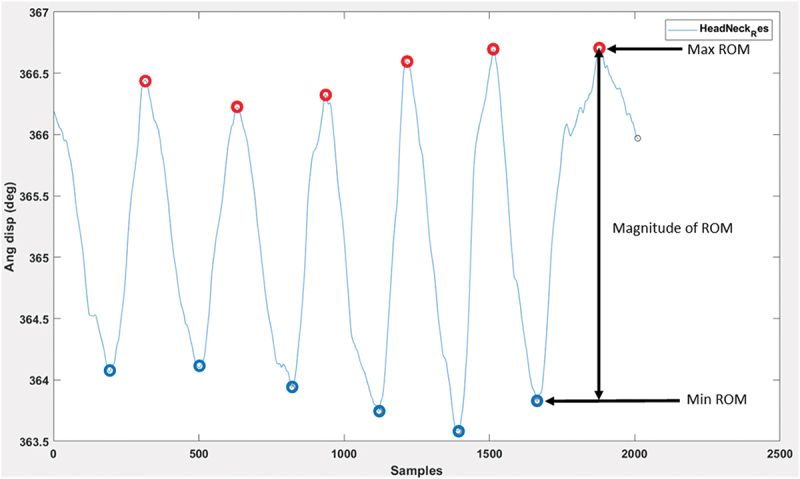
Example data set of ROM for six repetitions of movement. The y-axis displays the angular displacement of the head. The x-axis represents time (unspecified unit).

Cervical active ROM was considered as the magnitude from the neutral to the final position for each active movement. Xsens^TM^ raw data was exported to MVNX files then analysed using a customised MATLAB programme (https://www.mathworks.com/matlabcentral/fileexchange/126525-neckcare_xsens_analyser; R2022a; MathWorks, Natick, MA, USA). The Xsens^TM^ tracks cervicocephalic movement through two joints (i.e. C1–head and T1–C7), and to capture entire cervical motion we therefore opted to calculate a composite angle from both joints for data analysis. Peaks and valleys in the data were identified manually to determine the peak angle for each repetition for both the primary and associated planes of motion (Figure 3). This method of data extraction has demonstrated good inter-rater reliability (ICC 0.996) (English et al. 2023). Individual participant data was then exported to Microsoft Excel. All trials were evaluated using the video recording and invalid trials (i.e. whereby the laser exceeded the bullseye boundary) were excluded, allowing the remaining trials to be averaged for each movement.

### Statistical analysis

Statistical analysis was completed using the software JASP (version 0.18.1, 2023). Mean ROM and SDs from the Xsens^TM^ were collected for each cervical movement in the primary and associated planes of movement for each participant. These individual participant data were used to derive group primary plane mean ROM and was used to judge if the head-mounted laser was detecting movement in the primary plane. Because the method ensured that the maximum amount of movement recorded by the laser was 4.5° (i.e. trials that exceeded the radius of 4.5° were discarded), it was judged that the laser method is invalid if group primary plane mean ROM (Xsens^TM^) exceeded this range. A one-sample *t*-test or one-sample Wilcoxon test with a test value of less than 4.5° was employed to primary plane group mean data. Data were checked for normality using the Shapiro–Wilk test (Shapiro and Wilk 1965). A Student’s *t*-test was employed for normally distributed data and a Wilcoxon test for non-normally distributed data. A one-sided *p* value of 0.025 or less was deemed significant and indicative of validity of the head-mounted laser. Multiple comparison correction was not applied because analyses were hypothesis-driven, with a limited number of pre-specified comparisons. Movement variability between participants was measured using group SDs for each cervical movement, and variability within participants was assessed by evaluating the SD of ROM across repetitions for each movement (Bergin et al. 2014).

## Results

**Figure 4.**
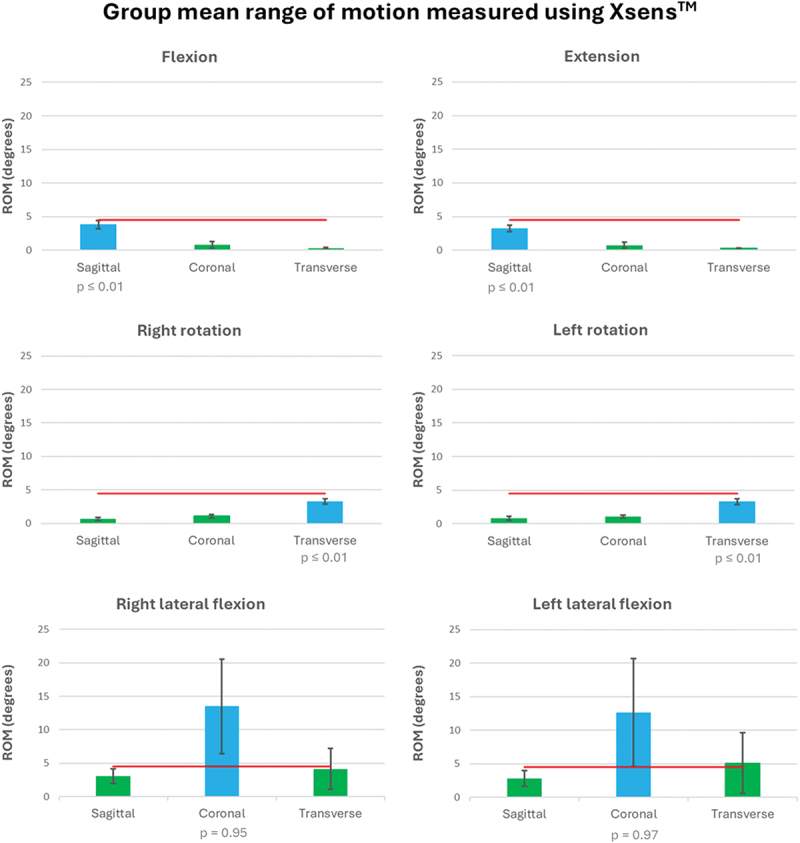
Group mean range of motion for primary (blue) and associated planes (green), measured using Xsens^TM^. Error bars represent ROM 95% confidence intervals. The horizontal red line corresponds to the 4.5° threshold used to judge validity.

The laser beam was retained within the bullseye for most trials, allowing most trials to be included in the analysis (total 442/504 = 88%, flexion 73/84 = 87%, extension 80/84 = 95%, right lateral flexion 75/84 = 89%, left lateral flexion 71/84 = 85%, right rotation 72/84 = 86% and left rotation 71/84 = 85%). The Shapiro–Wilk test indicated that the data were generally not normally distributed and therefore the Wilcoxon test was employed for analysis, except left rotation was normally distributed and the Student *t*-test was used in this instance. Group primary plane mean Xsens^TM^ ROM was within the 4.5° threshold for cervical flexion (mean 3.81, SD 1.18), extension (mean 3.22, SD 0.92), left rotation (mean 3.29, SD 0.80) and right rotation (mean 3.31, SD 0.76) movements, indicating appropriate measurement comparison of the laser method with the IMU (*p* ≤ 0.01) (Figure 4). However, left lateral flexion (mean 12.64, SD 15.01) and right lateral flexion (mean 13.48, SD 13.27) Xsens^TM^ ROM exceeded the 4.5° threshold, indicating that the laser does not capture all movement in the coronal plane (right lateral flexion *p* = 0.95; left lateral flexion *p* = 0.97). This may be explained by analysis of associated movements, which were small for cervical flexion and extension (mean Xsens^TM^ ROM range 0.26°−0.78°) and rotation (mean Xsens^TM^ ROM range 0.68°−1.08°), but there was increased associated rotation during lateral flexion (mean Xsens^TM^ ROM range 2.81°−5.01°). These results suggest that lateral flexion movement exceeded 4.5° because of concurrent associated rotation.

Movement variability was greater between participants than within participants, with all within-participant SDs smaller than between participant SDs across all movements and associated planes (Table 1). However, between-participant SDs were greatest for lateral flexion, indicating that participants utilised varying movement strategies during this particular motion.

**Table 1.** Movement variability measured by standard deviations between participants and within participants (N = 14).

Movement	Sagittal plane		Coronal plane		Transverse plane	
	Between N	Within N	Between N	Within N	Between N	Within N
Flexion	1.18°	0.61°	0.90°	0.18°	0.15°	0.12°
Extension	0.91°	0.65°	0.83°	0.15°	0.14°	0.14°
Right rotation	0.45°	0.32°	0.48°	0.36°	0.76°	0.55°
Left rotation	0.56°	0.54°	0.40°	0.39°	0.80°	0.51°
Right lateral flexion	2.05°	0.95°	13.28°	1.84°	5.65°	1.44°
Left lateral flexion	2.22°	0.75°	15.01°	1.65°	8.53°	1.01°

## Discussion

The head-mounted laser was appropriate for detecting cervical flexion, extension and rotation movements, with mean Xsens^TM^ cervical ROM falling within the 4.5° threshold. However, mean Xsens^TM^ right and left lateral flexion movement was 12.10° and 11.34° respectively, indicating that the head-mounted laser was unable to detect all coronal plane movements. Our study indicates that participants performed the movement consistently but there was a range of strategies between individuals in how to produce the lateral flexion movement whilst keeping the laser beam within the bullseye. This is illustrated by small standard deviations in ROM for individual participant movements (0.12°−1.84°) and larger standard deviations between participant mean ROM (0.14°−15.01°).

The head-mounted laser is commonly used to measure movement for cervicocephalic proprioceptive tests, and joint position error greater than 4.5° is considered clinically significant (Revel et al. 1991). Given the small differences between people with and without neck pain (de Vries et al. 2015; Stanton et al. 2016), there is a need to utilise highly accurate equipment to measure their sense of position. However, the primary finding of this study is that the laser does not capture all movements during lateral flexion. This was expected and is supported by other research which illustrates that lateral flexion is associated with concurrent rotation (Ferrario et al. 2002; Cook et al. 2006). During cervical lateral flexion and rotation, the oblique orientation of zygapophyseal joints creates complex multiplanar coupled motions (Ishii et al. 2006). Whilst both lateral flexion and rotation display coupling motions, coupling is comparatively less during rotation because of the C1–2 functional pivot joint which allows for cervical rotation in the transverse plane, particularly at the inner range of movement (Ogince et al. 2007). This could explain why there was minimal associated planar movement during rotation in this study, whereby movement was restricted to within 4.5° of the resting anatomical neutral position. In healthy people, the amount of contralateral rotation during full lateral flexion is quite large, comprising 18.0–23.6° (Ferrario et al. 2002). It is hypothesised that contralateral rotation occurring during lateral flexion is compensatory to facilitate a forward-facing visual field (Ishii et al. 2006). The theory is supported by this study which showed that participants were able to keep the laser within the 4.5° bullseye but reach 12.10° (right) and 11.34° (left) of mean lateral flexion.

Cervical ROM is important because it is inversely correlated with disability for people with chronic neck pain (Muñoz-García et al. 2016). Measurement of movement across all three planes is essential to accommodate for the heterogenous nature of patient presentations, where reductions in ROM can vary between directions (Lemeunier et al. 2018). It is therefore essential to identify subtle impairments in rotation and lateral flexion so that appropriate muscle groups can be targeted in rehabilitation programs (O’Leary et al. 2009). Generally, exercise rehabilitation for impaired joint position sense involves the clinician identifying the specific impairment, and then instructing the patient to repeatedly move from a start position to the target position (impaired position) using visual feedback to assist sensorimotor learning (Clark et al. 2015). In the cervical region, this is usually completed by asking the patient to move the head with the eyes closed to a specific position, and then opening the eyes to check accuracy (Clark et al. 2015). Application of these techniques using the head-mounted laser for cervical lateral flexion movements is difficult given the results of this study. When assessing and treating impairments in lateral flexion, clinicians may consider the use of alternative tools, such as IMU devices (Raya et al. 2018; English et al. 2023). Advances in technology have enabled the introduction of two-sensor and single-sensor IMU devices into the clinical setting, which can provide accurate biofeedback for clinicians and patients (Raya et al. 2018; English et al. 2023). In addition to measurement of position sense, such devices have the added capability of movement-sense analysis to enable thorough cervical proprioceptive assessment (Rosker et al. 2023). However, these positives need to be weighed against associated costs, accessibility and operator training needs. Generally, clinicians require that assessment methods are accurate, affordable and timely, and further studies should evaluate the feasibility of using IMU technology in a clinical setting.

This study found greater differences in movement variability between participants compared to within participants. Given that the tool used (Xsens^TM^) to measure movement has been shown to be reliable and valid, these findings suggest that individuals in this study performed movements consistently but there were differences between people. There is conflicting evidence regarding movement variability in clinical populations, with some reporting less variability in movement in people with neck pain (Alsultan et al. 2019) whilst others report higher variability in movement (Vogt et al. 2007). Different conclusions across studies are perhaps reflective of heterogeneity in equipment used (3D motion capture versus ultrasonic analysis) measurement methods (distance versus ROM versus axis of movement), and pain intensity and location. Variability of movement analysis indicates that participants in this study moved their head-and-neck in a consistent pattern, but there were differences in how people moved compared to one another. However, the sample size in this study was too small for robust statistical analysis in this respect. Future research should investigate movement variability between populations using standardised protocols and large samples. Additionally, further studies should establish the clinical applicability of single-sensor IMU devices by analysing test–retest reliability (intra-rater and inter-rater) for various proprioceptive tests including head to target, head to neutral and sense of movement protocols.

## Limitations

The sample size was potentially small, limiting generalisability and increasing the risk of a type two error. The analysis could have benefitted from a larger data set that would improve the confidence of findings, particularly regarding movement variability analysis. However, the sample size was sufficient to detect significant findings regarding the primary aim, providing confidence in these conclusions. The inclusion of healthy participants was considered appropriate, as the aim of this study was to identify whether the head-mounted laser could accurately measure cervical movements across three planes. However, confounding factors such as variations in posture, head orientation and proprioceptive ability may have affected the results of this study. Whilst screening was completed using subjective assessment by a physiotherapist, further screening using physical assessment may have identified potential impairments in participants (e.g. vestibular, joint stiffness etc.) which may have also influenced findings or provided greater context. Additionally, further studies should evaluate specific implications to clinical populations with particular focus on the head-to-neutral joint position error test and comparison of movement variability in people with neck pain versus healthy controls during full ROM. The current study involved performance of a novel task where ROM was constrained by the experimental procedure which only assessed a small amplitude in ROM, limiting the generalisability of findings regarding movement variability.

## Conclusions

Accurate measurement of cervical ROM is essential to identify potentially small but significant sensorimotor impairments in people with neck pain. This study found that the head-mounted laser is an appropriate clinical tool for measuring cervical flexion, extension and rotation ROM. However, some individuals performed a large amount of associated contralateral rotation during lateral flexion which impeded the ability of the laser to detect movement in the coronal plane properly. It is therefore recommended that clinicians employ alternative equipment to objectively measure cervical lateral flexion movement, particularly for sense of position tests. IMU technology is proven to capture movement across all three planes and is a good option. Future research should investigate the applicability of these and other suitable tools in the clinical practice setting and applied to people with neck pain.
